# Raynaud’s Secondary to Granulomatosis With Polyangiitis

**DOI:** 10.7759/cureus.17551

**Published:** 2021-08-30

**Authors:** Sai Swarupa Vulasala, Shohana Ahmed, Nirmal K Onteddu, Maneesh Mannem, Srikanth Mukkera

**Affiliations:** 1 Radiology, University of Florida College of Medicine – Jacksonville, Jacksonville, USA; 2 Internal Medicine, Texas Tech University Health Sciences Center – Permian Basin, Odessa, USA; 3 Internal Medicine, Flowers Hospital, Dothan, USA; 4 Division of Rheumatology, Texas Tech University Health Sciences Center, Odessa, USA

**Keywords:** granulomatosis with polyangiitis, sildenafil, bosentan, prostacyclins, emerging therapies, refractory raynauds, calcium channel blockers, wegener granulomatosis, raynaud phenomenon, secondary raynaud

## Abstract

Raynaud’s phenomenon (RP) is an episodic digital vasospastic condition that is prevalent among 5% of the population. The symptoms range from reversible pallor to ischemia and gangrene. RP can be primary or secondary. We discuss a case of severe RP secondary to granulomatosis with polyangiitis (GPA) that presented with ischemia and gangrene. Studies show that approximately <1% of GPA cases have similar presentations. Early diagnosis and management are essential to halt the progression of ischemia. Calcium channel blockers are the first-line medications used in RP. Phosphodiesterase type 5 inhibitors, endothelin receptor antagonists, and prostacyclin analogs are proven to be effective in cases of severe RP. Sympathectomy and amputation are considered as the extreme options in patients with refractory symptoms.

## Introduction

Raynaud’s phenomenon (RP) is an episodic vasospastic condition affecting the capillaries of distal extremities. RP is a complex of symptoms resulting from vascular compromise and is best described as a triad of pallor, cyanosis, and reactive hyperemia. The symptoms are exacerbated by exposure to cold temperatures or emotional stress. RP could be primary or secondary. Eighty to ninety percent of RP cases are identified as idiopathic/primary RP [1] with no underlying etiology. The secondary RP (SRP) has a broad etiology including connective tissue, hormonal, vascular, and hematological abnormalities. Our patient presented with digital ischemia and gangrene due to severe RP secondary to granulomatosis with polyangiitis (GPA). According to Lau et al., this presentation is one of the rare dermatological manifestations of GPA with a reported prevalence of approximately <1% [2]. In this case report, we discuss the clinical presentation and novel treatment of our patient along with some emerging therapies that are being studied for treating RP. 

This article was previously presented as a poster presentation at the 2021 Canadian Rheumatology Association (CRA) Annual Scientific Meeting, February 24-26, 2021.

## Case presentation

**Figure 1:**
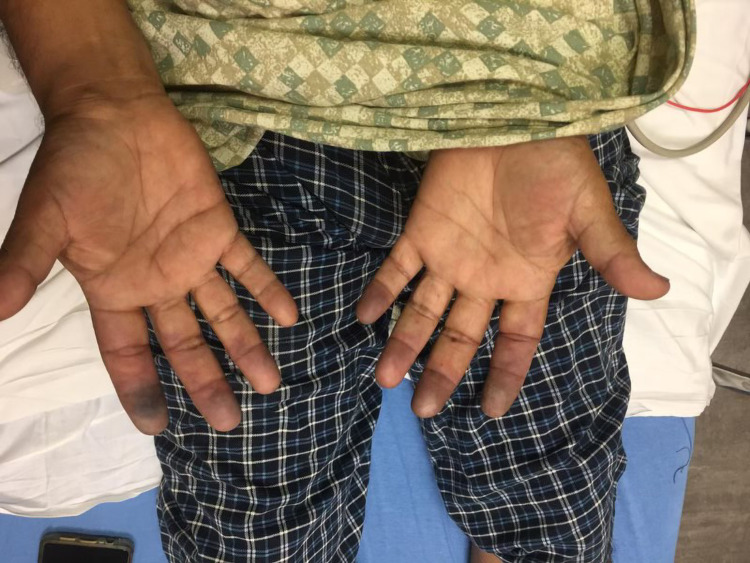
Cyanosed fingertips on the day of admission

**Figure 2:**
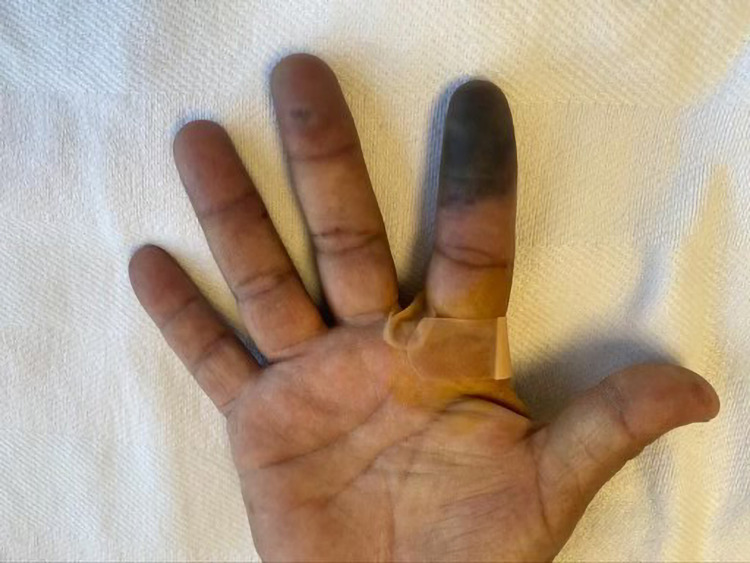
Progressive worsening of ischemia of right index finger

**Figure 3:**
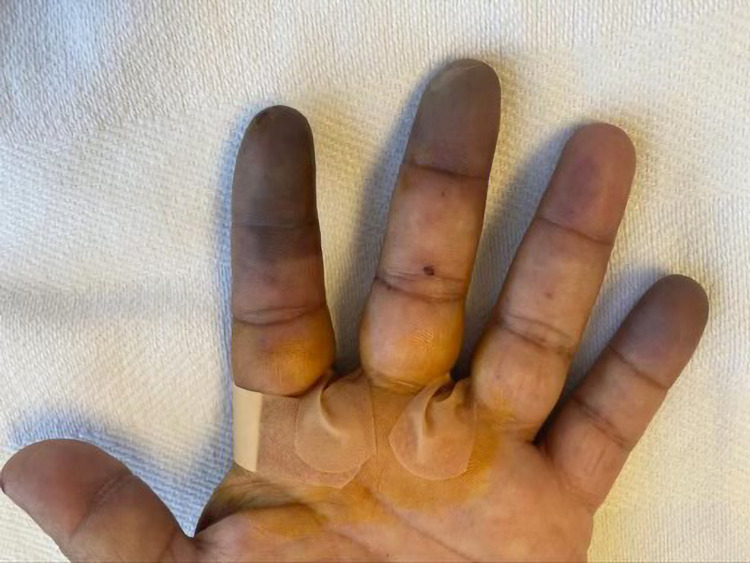
Progressive worsening of ischemia/active Raynaud’s phenomenon of left index, middle, and little fingertips

**Figure 4:**
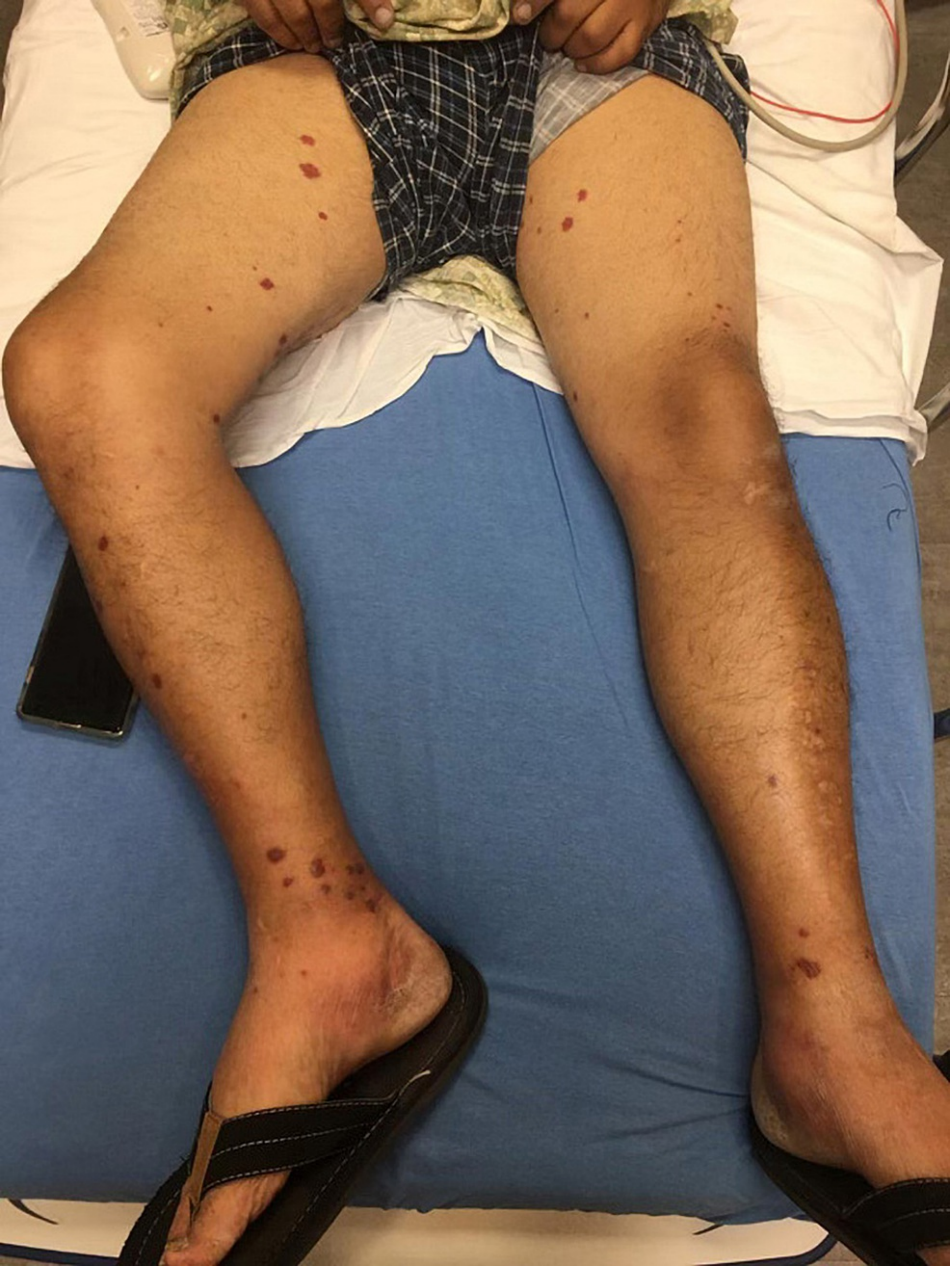
Purpura on bilateral lower extremities

A 51-year-old diabetic man presented with a two-week history of progressive pain, swelling, dynamic skin, and color changes involving bilateral fingertips. A review of systems was positive for a purpuric rash on the lower extremities and polyarthralgia. On examination, he was afebrile with a heart rate of 78/min, blood pressure 119/79 mm Hg, respiratory rate 18/min, and SpO_2_ 98% on room air. His fingers were swollen, tender, and his fingertips showed cyanosis in both hands without ulceration as demonstrated in Figures 1-3. There was palpable purpura on both legs extending up to the upper thigh as shown in Figure 4. Positive laboratory studies include erythrocyte sedimentation rate 68 mm/h, C-reactive protein 13.5 mg/dL, urine red blood cells 16 cells/high-power field, urine protein 70 mg/dL, D-dimer 8.07 ug/mL, rheumatoid factor >256 IU/mL, serum proteinase 3 IgG 298 AU/mL, and anti-neutrophil cytoplasmic antibody (ANCA) IgG by immunofluorescence assay <1:20. He tested negative for anti-nuclear antibody, anti-La/SSB (anti-Sjögren's-syndrome-related antigen B) antibody IgG, anti-Ro/SSA (anti-Sjögren's-syndrome-related antigen A) 52 antibody IgG, anti-Ro/SS-A 60 antibody IgG, Smith antibody IgG and Smith/ribonucleoprotein antibody IgG, cold agglutinins, cryoglobulin qualitative screen, human immunodeficiency viruses, hepatitis B & C, and tuberculosis quantiferon. The levels of C3 and C4 complements were normal. The prompt diagnosis and treatment are important in the long-term reduction of morbidity and mortality.

**Figure 5:**
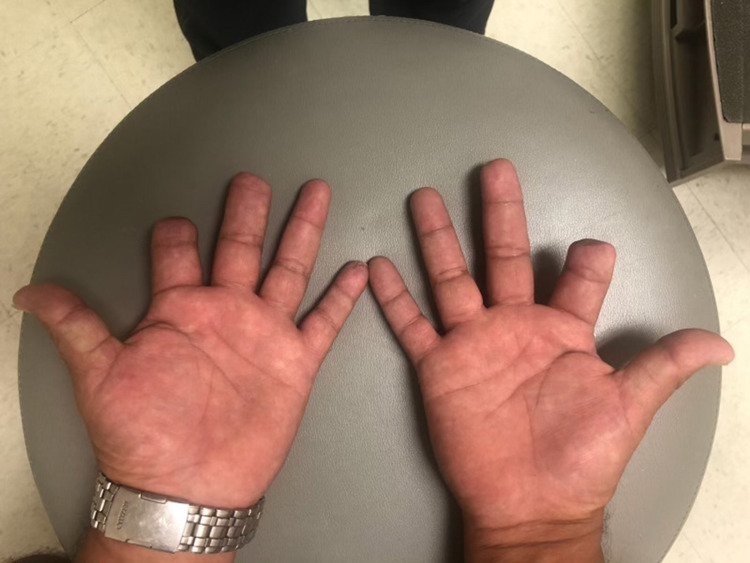
Post amputation of gangrenous digits left and right

For GPA, he was initiated on methylprednisolone and rituximab. Initial therapeutic regimens for RP included aspirin, nifedipine, topical nitroglycerin, and sildenafil. However, due to his progressive symptoms of active RP, the patient also required a digital nerve block of three ischemic digits along with the escalation of therapy with bosentan and epoprostenol. Despite timely therapy, the patient's finger ischemia worsened, and he developed dry gangrene in three fingers. After 12 days of remission induction treatment, his symptoms of purpura and digital cyanosis improved in the remaining fingers. The patient underwent an interval amputation of three digits (Figure 5). He was sent home on remission maintenance therapy with methotrexate for GPA and nifedipine, sildenafil along with bosentan for RP.

## Discussion

The phenomenon of vasospasm on exposure to cold is a reversible physiologic entity and is first described by Raynaud. The level of response varies between any two individuals depending on the cold tolerability of the individual and the degree of sympathetic activity [3]. RP is considered pathological when associated with symptoms of ischemia and followed by complications like ulcers, gangrene, and amputation.

RP results from an imbalance between vasoconstrictors like endothelin (ET), and vasodilators like nitric oxide and prostacyclin [4]. With the progressive imbalance, the fibrotic growth factors such as transforming growth factor-beta promote the remodeling of vessels resulting in obliterative vasculopathy. The characteristic triad as a consequence of hypoxia constitutes White (vasoconstriction)→Blue (cyanosis)→Red (reactive hyperemia). The triphasic response is rare to be appreciated in all the patients. Hence, biphasic color change with pallor as the requisite component can be considered in diagnosing RP. The prolonged RP leads to digital ischemia and thereby causing pain and numbness in the extremities. RP can be classified into primary and secondary. The primary RP carries no underlying etiology and is observed in individuals with risk factors such as female sex, low body mass index, smoking, and atherosclerosis. The SRP is possibly a manifestation of underlying etiologies including rheumatological, neurological, and hematological disorders (Table 1). The complications are more severe in SRP ultimately leading to amputation if not treated immediately [1].

**Table 1:** Secondary causes of Raynaud's phenomenon

Secondary etiologies of Raynaud’s	
Rheumatologic etiologies	Systemic sclerosis; Sjogren’s syndrome; mixed connective tissue diseases; systemic lupus erythematosus; vasculitis - small, medium, and large vessel
Neurological etiologies	Thoracic outlet obstruction; carpal tunnel syndrome
Hematological etiologies	Cryoglobulinemia; paraproteinemia; IgM cold agglutinin disease
Endocrinological etiologies	Hypothyroidism
Vascular etiologies	Atherosclerosis; thromboangiitis obliterans

**Figure 6:**
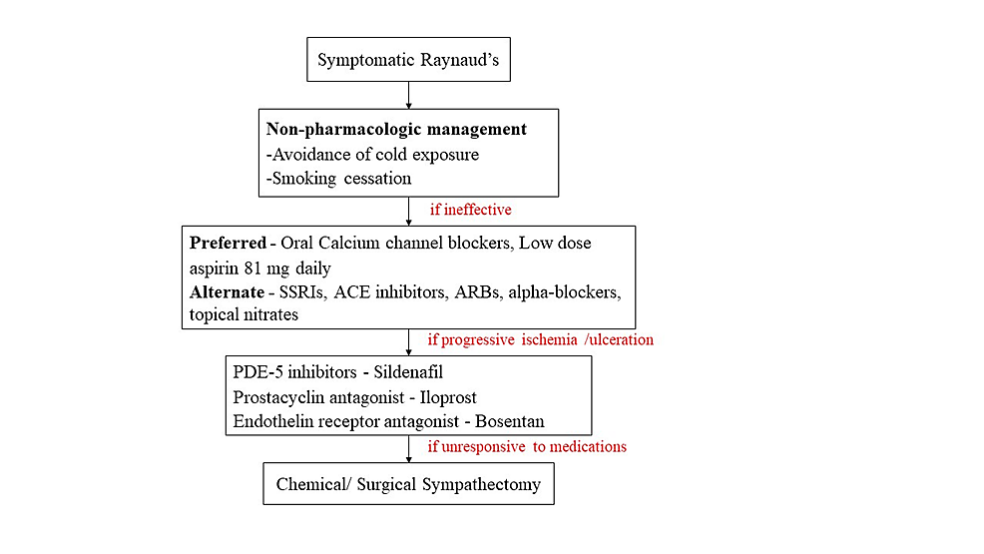
Escalation therapy for Raynaud’s phenomenon SSRIs, selective seratonin reuptake inhibitors; ACE, angiotensin-converting enzyme; ARBs, angiotensin receptor blockers; PDE-5, phosphodiesterase type 5.

Our patient presented with RP secondary to GPA, an autoimmune small-vessel vasculitis. GPA was first described by a German doctor in the late 19th century. It is a necrotizing vascular inflammation secondary to targeted autoantibodies C-ANCA (cytoplasmic antineutrophil cytoplasmic antibody) and P-ANCA (perinuclear antineutrophil cytoplasmic antibody). C-ANCA is directed against proteinase-3 in the granules of neutrophils, whereas P-ANCA is directed against myeloperoxidase in neutrophils. The sensitivity and specificity of ANCA to GPA are 66% and 98%, respectively [5]. GPA is associated with c-ANCA in 80% of cases [6] and P-ANCA in less than 20% of cases [7]. The pathological hallmarks of GPA include systemic necrotizing vasculitis, necrotizing inflammation, and necrotizing glomerulonephritis [6]. The resulting vasculitis is responsible for the occlusion of vessels, which results in ischemia of the tissues. The clinical spectrum of GPA is wide, making it challenging to diagnose. Respiratory tract and renal manifestations are classic to GPA, ranging from sinusitis (58%) to respiratory failure (6%) and glomerulonephritis (51%) to renal failure (33%) [8]. The skin involvement in GPA is rare and is reported in only 20% of patients with palpable purpura as the most common symptom [8]. Diagnosis of GPA is based on clinical manifestations, positive ANCA serology, and histological evidence. As there are immense clinical trials and substantial data regarding definitive management of GPA, we concentrated on conferring the treatment of RP related to GPA. The stepwise proposed escalation of therapy for RP along with the mechanism of actions and side effects is described in Figure 6 [9] and Table 2, respectively.

**Table 2:** Medications that are found effective in Raynaud’s phenomenon RP, Raynaud's phenomenon; PDE-5, phosphodiesterase type-5; cGMP, cyclic guanosine monophosphate; NO, nitric oxide;  PGI2, prostaglandin I2; ERA, endothelin receptor antagonists

Class of medication	Mechanism of action	Effect of medication	Adverse effects
Calcium channel blockers	Decrease intracellular calcium content	Decrease the severity and frequency of RP attacks	Hypotension, flushing, peripheral edema
Aspirin	Inhibits platelet aggregation through thromboxane A2 blockage	Improves the digital microcirculation	Gastric mucosal damage and bleeding; hypersensitivity; intracranial bleed
PDE-5 inhibitors	Inhibits cGMP degradation; increases the NO levels	Decreases the duration and frequency of RP attacks	Headache, muscle pain, rhinorrhea, visual abnormalities
Prostanoid therapy	Vasodilatory effects of PGI2	Decreases the severity and frequency of attacks; heals digital ulcers	Flushing, headache
ERA	Antagonizes the effect of endothelin; vasodilation	Reduces the incidence of new ulcers	Elevated transaminases, palpitations, headache

Non-pharmacological management is recommended in patients with mild symptoms and includes wearing adequate clothing to prevent heat loss from the body, avoiding smoking, reconsidering medications such as beta-blockers triggering digital vasoconstriction.

Dihydropyridine calcium channel blockers (CCBs) are the first-line medications in the treatment of both primary and secondary RP. They decrease the calcium content in the cell thereby causing vasodilation. CCBs are found to be effective in reducing the severity and frequency of RP attacks when given in higher doses compared to lower doses [10]. Common side effects of CCBs include flushing, hypotension, headache, and peripheral edema. Topical nitrates, pentoxifylline, selective serotonin reuptake inhibitors (fluoxetine), alpha-blockers (prazosin), angiotensin receptor blockers (losartan), and angiotensinogen-converting enzyme inhibitors (quinapril, enalapril) can be used in patients intolerant to CCBs [11]. However, their efficacy has not been studied yet.

Phosphodiesterase type-5 (PDE-5) inhibitors like sildenafil, endothelin receptor antagonists (ERAs) like bosentan, and prostaglandin I2 (PGI2) analogs like epoprostenol are recommended in patients refractory to CCBs and behavioral modifications.

PDE-5 inhibitors exhibit a therapeutic effect by inhibiting the degradation of cGMP (cyclic guanosine monophosphate). cGMP is a vasodilator and exerts its effect by increasing the levels of nitric oxide (NO) and decreasing the intracellular calcium. A meta-analysis by Roustit et al. [12], reported that PDE-5 inhibitors benefit in terms of reducing the duration and frequency of RP attacks. Sildenafil, vardenafil, udenafil, and tadalafil are some of the known PDE-5 inhibitors. Sildenafil is a short-acting drug (T½ 4 hours) and is recommended in treating acute presentations, whereas tadalafil is a longer-acting drug that showed efficacy as an add-on therapy to CCBs. It prevented new ulcers along with the healing of existing ulcers [13]. Side effects include headache, muscle pain, visual abnormalities, flushing, dizziness, and hypotension.

ERAs act by antagonizing the effect of vasoconstrictor, ET. ET is secreted by endothelial cells and causes vascular smooth muscle contraction. The systematic literature review by Garcia et al. including three randomized control trials concluded that bosentan reduced the incidence of new ulcers; however, it does not aid in the healing of already existing ulcers [9,11]. The side effects of bosentan include elevation in transaminases, erythema, flushing, and palpitations [14]. These are improved with the reduction of bosentan dosage.

Prostanoids such as PGI2 cause vasodilation and inhibit platelet aggregation. Intravenous (IV) epoprostenol is a Food and Drug Administration-approved medication in treating patients with RP that is not responding to conventional therapy (CCBs, topical nitrates, behavioral modifications) [15]. However, it is contraindicated in patients with congestive heart failure due to left ventricular dysfunction and drug hypersensitivity. The common adverse effects of epoprostenol include pulmonary edema, catheter infections, and bleeding. Therefore, the patient should be monitored cautiously [15]. In a meta-analysis by Bionka et al., oral and IV iloprost decreased the severity of RP attacks by >50% compared to placebo [10]. Prostanoids also decrease the frequency of attacks and heal digital ulcers [9]. Bionka et al. did not find any significant evidence of IV alprostadil in the treatment of SRP [10]. 

Low-dose aspirin may be considered as antithrombotic prophylaxis and to improve microcirculation in patients with severe RP such as digital ulcerations and gangrene. However, the efficacy has not been studied extensively [9].

Sympathectomy and amputation are advised as the extreme option in cases of irreversible tissue necrosis. Digital periarterial sympathectomy and endoscopic thoracic sympathectomy can be done in patients with refractory digital ischemia and severe complications [16].

Our patient was initially treated with conventional therapy including nifedipine, aspirin, and topical nitroglycerin. Later, with progressive symptoms, the gradual escalation of therapy was done with sildenafil, bosentan, and epoprostenol along with the digital nerve block and amputation of gangrenous digits.

Emerging therapies

Calcitonin gene-related peptide (CGRP) is a vasodilator and studies [17] show that there may be deficiency of CGRP in the digits resulting in vasoconstriction and RP attacks. The infusion of CGRP improved the blood flow to digits and reduced the digital ulceration. Also, the treatment of migraine in RP patients with CGRP antagonists was associated with digital ischemia and ulceration. However, trials need to be conducted to understand the efficacy of CGRP in RP attacks. In studies by Huntgeburth et al. [18] and Ruaro et al. [19], riociguat (soluble guanylate cyclase stimulator) and aminaphtone (a derivative of 4-aminobenzoic acid), respectively, are proven to be effective in improving the digital blood flow yet warrant further trials. Microemulsion containing nitroglycerin gel (MQX-503) is being studied as a topical alternative averting systemic side effects [20].

## Conclusions

The GPA presenting as severe RP is a very rare phenomenon. In such circumstances, patients should be initially started on CCBs along with GPA-specific management. This article focused on the escalating therapy of refractory RP with PDE-5 inhibitors, prostacyclin analogs, and ERAs. Sympathectomy or amputation is considered in unresponsive and progressing symptoms. 
